# Comparison of the Factor Structure of the Patient Health Questionnaire for Somatic Symptoms (PHQ-15) in Germany, the Netherlands, and China. A Transcultural Structural Equation Modeling (SEM) Study

**DOI:** 10.3389/fpsyt.2018.00240

**Published:** 2018-06-26

**Authors:** Rainer Leonhart, Lars de Vroege, Lan Zhang, Yang Liu, Zaiquan Dong, Rainer Schaefert, Sandra Nolte, Felix Fischer, Kurt Fritzsche, Christina M. van der Feltz-Cornelis

**Affiliations:** ^1^Department Social Psychology and Methodology, Institute of Psychology, University of Freiburg, Freiburg, Germany; ^2^Clinical Centre of Excellence for Body Mind and Health, GGz Breburg, Tilburg, Netherlands; ^3^Department Tranzo, Tilburg School of Social and Behavioral Sciences, Tilburg University, Tilburg, Netherlands; ^4^Mental Health Center, West China Hospital of Sichuan University, Chengdu, China; ^5^Division of Internal Medicine, Department of Psychosomatic Medicine, University Hospital Basel, Basel, Switzerland; ^6^Department of General Internal Medicine and Psychosomatics, University of Heidelberg, Heidelberg, Germany; ^7^Faculty of Medicine, University of Basel, Basel, Switzerland; ^8^Department of Psychosomatic Medicine, Center for Internal Medicine and Dermatology, Charité - Universitätsmedizin Berlin, corporate member of Freie Universität Berlin, Humboldt-Universität zu Berlin, and Berlin Institute of Health, Berlin, Germany; ^9^Population Health Strategic Research Centre, School of Health and Social Development, Deakin University, Burwood, VIC, Australia; ^10^Department of Psychosomatic Medicine and Psychotherapy, Faculty of Medicine, Medical Center - University of Freiburg, Freiburg, Germany

**Keywords:** somatic symptoms, patient health questionnaire-15, factor structure, structural equation modeling (SEM), transcultural

## Abstract

**Background:** Persistent somatic symptoms are associated with psychological distress, impaired function, and medical help-seeking behavior. The Patient Health Questionnaire (PHQ)-15 is used as a screening instrument for somatization and as a monitoring instrument for somatic symptom severity. A bifactorial model has been described, with one general factor and four orthogonal specific symptom factors. The objective of the present study was to assess and to clarify the factor structure of the PHQ-15 within and between different countries in Western Europe and China.

**Method:** Cross-sectional secondary data analysis performed in three patient data samples from two Western European countries (Germany *N* = 2,517, the Netherlands *N* = 456) and from China (*N* = 1,329). Confirmatory factor analyses (CFA), and structural equation modeling (SEM) analysis were performed.

**Results:** The general factor is found in every sample. However, although the outcomes of the PHQ-15 estimate severity of somatic symptoms in different facets, these subscales may have different meanings in the European and Chinese setting. Replication of the factorial structure was possible in the German and Dutch datasets but not in the dataset from China. For the Chinese dataset, a bifactorial model with a different structure for the cardiopulmonary factor is suggested. The PHQ-15 could discern somatization from anxiety and depression within the three samples.

**Conclusion:** The PHQ-15 is a valid questionnaire that can discern somatization from anxiety and depression within different cultures like Europe or China. It can be fitted to a bifactorial model for categorical data, however, the model can only be recommended for use of the general factor. Application of the orthogonal subscales in non-European samples is not corroborated by the results. The differences cannot be ascribed to differences in health care settings or by differences in concomitant depression or anxiety but instead, a cultural factor involving concepts of disease may play a role in this as they may play a role in the translation of the questionnaire. Further research is needed to explore this, and replication studies are needed regarding the factorial structure of the PHQ-15 in China.

## Background

### Somatic symptoms, emotional distress, and disability

In everyday life, somatic symptoms are common causes of outpatient medical visits (1, 2). Patients with multiple distressing somatic symptoms present themselves in a variety of health care settings, such as primary, secondary and tertiary patient-centers (3–6). Poor self-rated health of patients was found to be associated with multiple somatic symptoms in Europe and in China (7–10). The number of somatic symptoms correlates well with impaired function and medical help-seeking behavior even after controlling for mental disorders (5). The number of somatic symptoms differs only slightly between patients with somatic symptoms explained by a medical disease and patients with unexplained somatic symptoms (11, 12). This indicates that the suffering of a patient with medically unexplained somatic symptoms should be taken seriously. In Western countries, a high number of somatic symptoms is associated with higher psychological distress, more functional impairment, higher disability, more health care utilization and a reduced quality of life (13–15). Given the large burden of somatic symptoms, especially unexplained symptoms that are currently not taken sufficiently seriously, the assessment of somatic symptoms and the analysis of the different facets of somatic symptoms is a useful and necessary part of every medical diagnostic process.

#### The patient health questionnaire (PHQ)-15

The Patient Health Questionnaire (PHQ)-15 (16) is a short, practical self-rating instrument for the screening of somatic symptoms. The PHQ-15 has also been suggested by the DSM-5 Workgroup on Somatic Symptom Disorders (SSD) as a measurement tool of somatic symptom severity for the classification of SSD (17, 18). The PHQ-15 was initially validated in primary care and a general hospital setting in the USA (16). Although usually the sum score is simply used as a measure for symptom load, several studies (16, 19–21) suggest that the different somatic symptoms in the PHQ-15 can be divided and bundled into four groups: cardiopulmonary, gastrointestinal, pain, and fatigue/general symptoms.

Witthöft et al. (22) assessed the underlying structure of the PHQ-15 in two datasets of college students (Germany, *N* = 1,520; Switzerland*, N* = 3,053). They conducted a confirmatory factor analysis assuming a bifactor model (1 general and 4 orthogonal specific symptom factors; gastrointestinal, fatigue, cardiopulmonary, and pain symptoms). Correlations with general and lower-order latent factors of depression, general somatic symptom distress, and health anxiety were found. These factors explain up to nearly 70% of the variance in the general somatic symptom factor. However, it remains to be shown if this structure also applies to other samples. The original validation of the PHQ-15 was in general hospital patients and primary care patients (16), but not in patients in specialty mental health institutions, so it would be useful to explore the factor structure in other health care settings such as the specialty mental health care setting.

Also, it is unclear whether the factor structure of the PHQ-15 questionnaire is comparable between samples from Europe and samples from other continents and cultures such as Asia, in particular China, since the cultural background shapes the interpretation of somatic symptoms and thereby influences an individual's illness perception and illness behavior (23, 24). In the literature, the influence of culture on experiencing somatic as well as psychosomatic symptoms has been explored. Evidence exists that cultural and personal explanatory models can contribute to the symptomatology of medically unexplained symptoms (25). Efforts have been made to make epidemiological research into this domain possible (26) and the need for appropriate instruments to assess physical symptoms in the context of distress in Asian cultures has been expressed (27).

Historically, there has been a popular belief that Asians manifest a lower prevalence of mood and anxiety disorders than their Western counterparts because they are more prone to experiencing and manifesting distress via somatic pathways (28–30). Among Chinese patients receiving psychiatric services, somatic symptoms such as pain, insomnia, and fatigue have been associated with depressive and anxiety disorders (31).

The PHQ-15 has been translated into many other languages and has been examined in samples from many other countries, e.g., Saudi Arabia, Spain, and Korea (32–34). This offers the potential for comparisons between different ethnic, cultural or geographical groups. However, so far, no studies have explored the factor structure in the context of different health care settings and West European vs. Chinese culture.

Another point of discussion concerns somatization. Somatization is defined as the tendency to experience and to express physical distress and symptoms that cannot be explained by pathological findings, to attribute them to a medical condition, and to seek medical care for them (35). It has also been described as a term related to our conceptualisation of disease, that is, to the tendency of doctors to attribute symptoms either to physically explainable medical conditions, or as medically unexplained symptoms (36). It has been suggested that physical symptoms occurring in the context of somatization may also concur with depression or anxiety (37). Hence, somatization, as assessed by PHQ-15, might be related to anxiety or depression as assessed by GAD-7 and PHQ-9, as somatization, anxiety, and depression are supposed to be related. It was hypothesized that this might be the case in China, as somatization has been assumed to be more common in China as a manifestation of underlying anxiety and depression (38). Hence, the association between scores on the PHQ-15 and GAD-7 and PHQ-9 should be explored in these different cultures.

### Rationale and aims of the study

In this study, we compare patients with SSD including all kinds of psychosomatic illnesses in a convenience sample from three different countries, namely Germany, the Netherlands, and China, that enabled us to compare three different health care settings, namely the general hospital inpatient psychosomatic setting (Germany), the specialty mental health outpatient setting providing treatment for SSRD patients with high complexity levels in terms of diagnostic problems, treatment issues and social challenges (8), in the Netherlands; and the general hospital (China) for SSD. Finally, this way we could compare two different cultures, namely the Western European and the Chinese culture.

The objective of the present study was to assess and to clarify the factor structure of the PHQ-15 within and between different countries.

We aimed to answer the following research questions:

1. Can the proposed bifactor model of Witthöft et al. (22) be replicated in three different study populations?
2. If not—are there more appropriate models within the three study populations?
3. Are there indications for cultural differences?
4. Is there an association between PHQ-9 and GAD-7 scores and the Witthöft factorial structure of the PHQ-15? More specifically, is there an association between PHQ-9 and GAD-7 scores and the potential general factor and/or the specific factors of the PHQ-15?

## Methods

### Study design

This is a cross-sectional, secondary analysis of data collected within several research projects or routine clinical care in the three countries. In Germany, the data were collected as part of routine clinical care. In China, the data were collected as part of a cross sectional study (39) in the general hospital setting. In the Netherlands, the data were collected as part of routine clinical care in the Clinical Centre of Excellence of Body Mind and Health (8, 40–42).

### Assessment instruments

#### PHQ-15

The PHQ-15 is a self-administered somatic symptoms subscale derived from the full PHQ (14, 43). The PHQ-15 was originally validated by Spitzer et al. (16). It includes 15 prevalent somatic symptoms that represent over 90% of the symptoms observed in primary care (16). The patients are asked to rate the severity of their symptoms during the previous 4 weeks on a 3-point scale as either 0 (“not bothered at all”), 1 (“bothered a little”) or 2 (“bothered a lot”). The questionnaire demonstrated good internal consistency [Cronbach's alpha = 0.80; (16)] and high relevance to symptoms. It is available in multiple languages.

#### Germany

The German translation of the PHQ-15 has been shown to have sound psychometric properties (44) and reference data from the general population is available (45). The PHQ-15, as well as the PHQ-9 and the GAD-7, were collected within the routine psychometric assessment of outpatients in the Department of Psychosomatic Medicine. Data analyzed here has been collected between January 2005 and February 2009. If patients were assessed multiple times, we included the first occasion only. Clinical diagnoses were made by trained physicians and psychologists. Along with questionnaires and clinical diagnoses, we collected sociodemographic data.

#### China

The validity and reliability of the Chinese version of the PHQ-15 (46) were examined in the general population of Hong Kong. The Hong Kong version of the PHQ-15 exhibited satisfactory internal consistency (Cronbach's alpha = 0.79) and stable 1-month test-retest reliability. Somatic symptom severity was positively associated with functional impairment and health service use. In Mainland China, the validity and reliability of the PHQ-15 were tested in the outpatient clinics of general hospitals in Shanghai (47). Cronbach's alpha was 0.73, and the test-retest reliability coefficient was 0.75 (10). There were moderate positive correlations between the PHQ-15 score and anxiety and depression values.

##### Translation of the PHQ-15

Three native Chinese speakers who resided in Germany updated the Chinese version of the PHQ-15 taking findings from recent research into account. The translation was based on the former Chinese version, which was translated from English to Mandarin (47). The differences to this former version include changes in item 1 and item 8: We changed “stomach pain” (胃痛或肚痛) in item 1 to “stomach and abdominal pain” (胃痛), in accordance to the suggestions of Lee, Ma (46). In item 8, “fainting spells” was replaced with “brief fainting” [短倒 (Cantonese), 短时间晕倒 (Mandarin)]. The Mandarin version of the PHQ-15 used in this study is available in the Appendix (Supplementary Material).

The data analyzed here were taken from a cross-sectional survey under routine clinical conditions. Participants in this study were inpatients recruited from different departments (e.g., oncology, cardiology, respiratory medicine, rehabilitation, geriatrics and gerontology, general practice, pain management, thyroid and breast surgery, rheumatology, and hepatic surgery). The validation of the PHQ-15 is a component of a larger project investigating the prevalence and recognition of inpatients with emotional distress and their treatment needs at a general hospital.

### Ethics statement

All patients were informed at intake that the Patient Related Outcome Monitoring (PROM) data pertaining to their treatment could be used on an anonymous basis for research, and were provided the opportunity to object to that during intake or anytime during treatment. PROM data of patients objecting to this were not used in this study. The study was approved by the ethics committees of the Shanghai Dong Fang hospital (XZ), the University Medical Centre Freiburg (KF), and GGz Breburg (CFC).

#### The netherlands

In the Netherlands, the PHQ-15 was applied in several research projects in primary care (48, 49), occupational health care (50), and the general hospital setting (51). In primary care, the PHQ-15 showed limited results in terms of validity (52) as it was found to have a tendency to be falsely positive for somatoform disorder, as it does not discern unexplained physical symptoms from explained physical symptoms. In occupational health care, validity was also limited both in terms of sensitivity as well as in terms of specificity (53). It is currently regularly applied in the intake procedure of the Clinical Centre of Excellence for Body, Mind, and Health (Dutch abbreviation: CLGG), which is a specialty mental health institution for patients with somatic symptom and related disorders. Data from this patient group is included in this comparative study. Before intake, patients at CLGG receive questionnaires using Routine Outcome Monitoring. Data for this study were collected during 2010 and 2016. A total of 465 patients filled in the PHQ-15, GAD-7, and PHQ-9.

#### Depression scale PHQ-9

This instrument assesses each of the nine DSM-IV depression criteria on a scale of “0” (not at all) to “3” (nearly every day) (13). The PHQ-9 demonstrated acceptable psychometric properties for the screening of patients with late-life depression. In the Netherlands, the PHQ-9 was validated in the Depression Initiative, a national study aimed at improving mental health care for people with depressive symptoms. It proved to be a feasible and valid instrument to screen for depressive disorder, to assess severity and to assess change in scores over time in the primary care, occupational health care and general hospital outpatient setting. It has also been proven to be feasible and valid for use in electronic monitoring systems in the context of blended mental health care in the occupational health setting. The PHQ-9 is a widely used and valid instrument in the Netherlands as well as in Germany (54–56).

In Chinese, within a primary care setting, this questionnaire showed a sensitivity of 0.86 and a specificity of 0.77 for depression screening (57).

#### Anxiety scale (GAD-7)

A seven-item anxiety scale (GAD-7) was used to assess the severity of generalized anxiety (58). In a German validation study (59), it has been shown to be a reliable and valid measure of anxiety in the general population. In a study in the Dutch primary Care setting, it showed limited validity as a screener and better validity as a case-finding instrument for general practitioners (60) and it proved to be feasible in the application of collaborative care in the primary care setting. In a Chinese general hospital population, this instrument showed good reliability and good criterion, construct, factorial, and procedural validity (23).

#### Statistical analyses

Using IBM SPSS (24.0) (61), and MPlus (62) 7.4 software, all three samples were analyzed using confirmatory factor analyses (CFA) and structural equation modeling (SEM). As the items of all questionnaires were categorical ordered the analyses were conducted with the robust weighted least squares estimator with a mean and variance adjusted test statistic (WLSMV). Because the chi-square test is sensitive to potentially irrelevant deviations from the implied model structure given large samples, we used established fit measures to evaluate the models. For the absolute fit, we chose the root mean square error of approximation (RMSEA). The comparative fit index (CFI) and the TLI (Tucker–Lewis Index) are reported as incremental fit indices. Following the advice of Hu and Bentler (63) RMSEA values lower than 0.06 and CFI/TLI values higher than 0.95 are considered as indicators of a good model fit. All coefficients (i.e., factor loadings and path weights) are reported as standardized coefficients.

**Figure 1:**
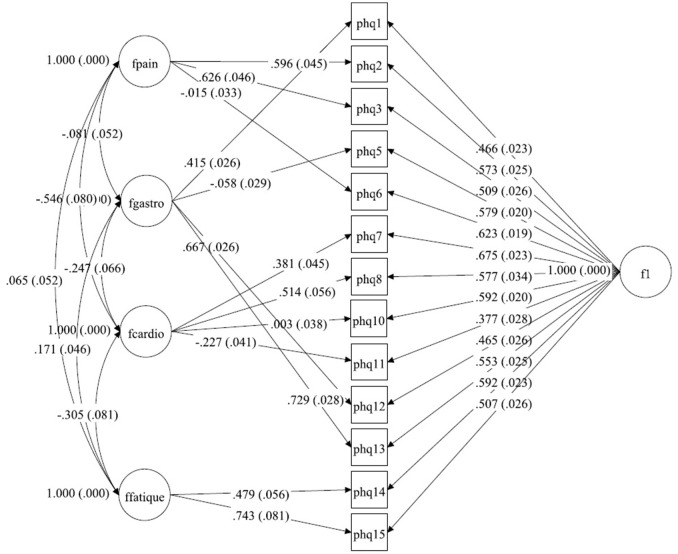
A bilateral model of somatic symptoms in the PHQ-15 in Germany, *N* = 2,517. With standardized factor loadings and standard error in parenthesis. Circle represents latent variables, squares refer to manifest variables, single headed arrows represent factor loadings, double headed arrows represent correlations.

**Figure 2:**
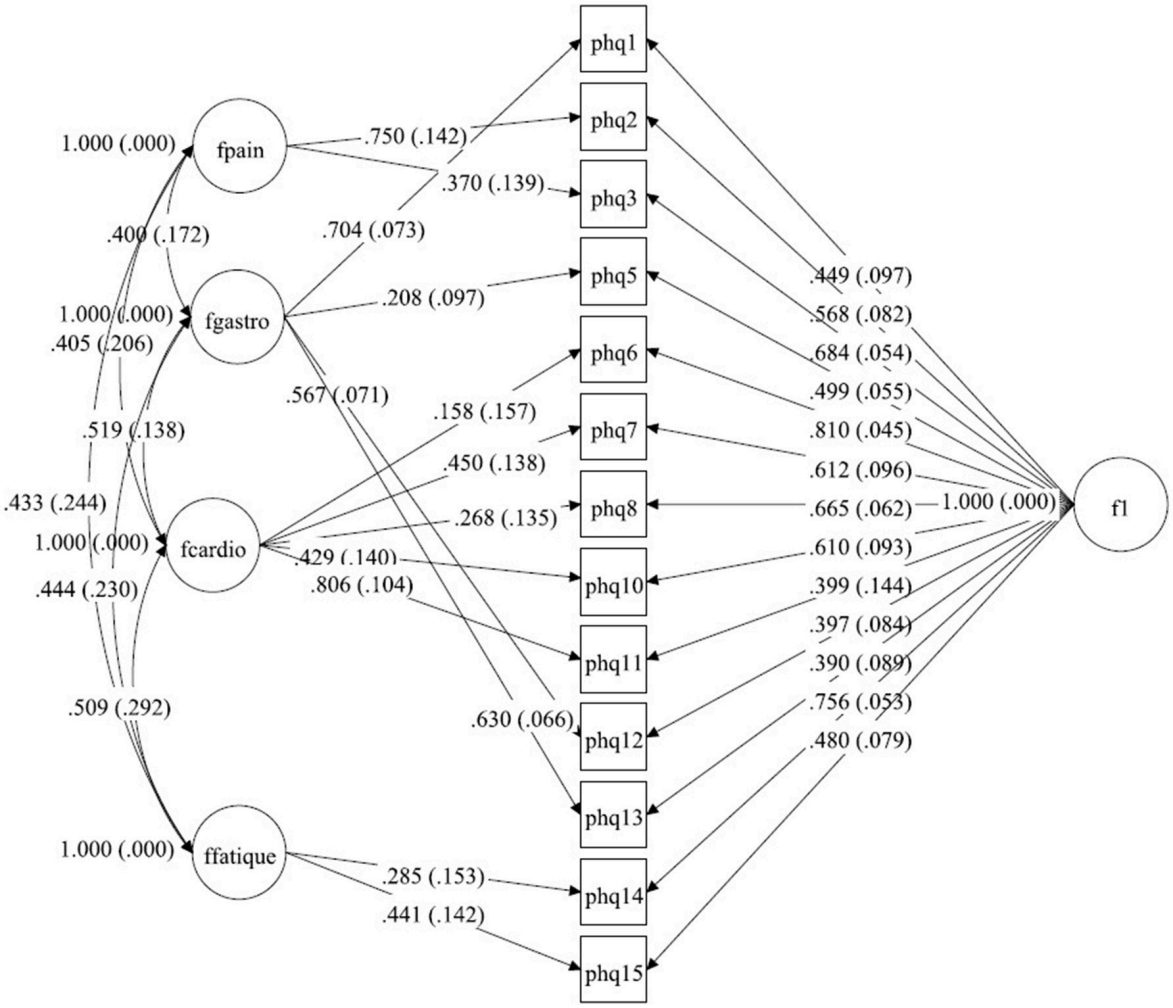
A bilateral model of somatic symptoms in the PHQ-15 in Netherlands, *N* = 456.With standardized factor loadings and standard error in parenthesis. Circle represents latent variables, squares refer to manifest variables, single headed arrows represent factor loadings, double headed arrows represent correlations.

**Figure 3:**
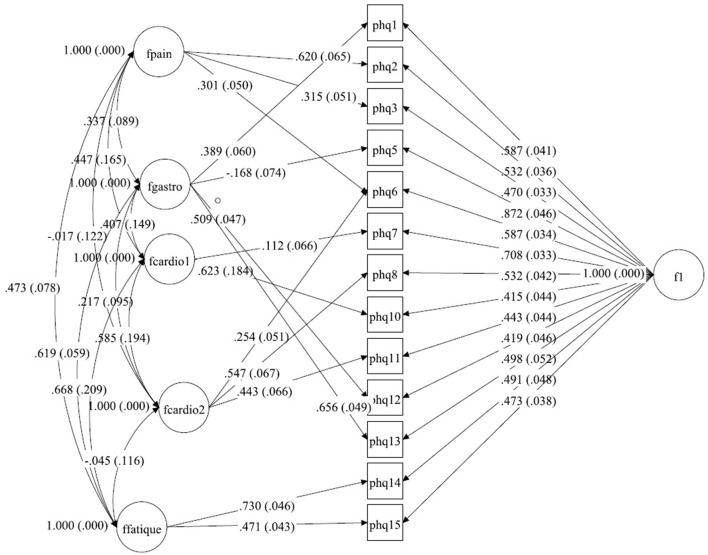
A bilateral model of somatic symptoms in the PHQ-15 in China, *N* = 1,329. With standardized factor loadings and standard error in parenthesis. Circle represents latent variables, squares refer to manifest variables, single headed arrows represent factor loadings, double headed arrows represent correlations.

Several CFA models were tested. In a first step, the bifactor model from Witthöft, Fischer (22) was estimated within a multigroup design. Because model fit was only acceptable, alternative models were sought in a second step. In this second step, the bifactor model [see Figure 1 (Germany), Figure 2 (Netherlands), and Figure 3 (China)] was estimated within each group and modification indices were assessed to improve fit indices over abovementioned cutoffs. In a third step, SEMs consisting of measurement models and a structural model was computed in order to determine the size of associations between the latent factors implied by the bifactor model and other relevant constructs (GAD-7 and PHQ-9).

## Results

### Description of the sample

Age and gender in the three samples are shown in Table 1.

**Table 1:** Demographic characteristics.

		**Germany**	**China**	**Netherlands**	***F*/Chi*2***	***df***	***p***	***R*^2^**
Total	*N*	2,517	1,322	456				
Age	Mean	42.56	53.76	41.02	259.68	*df*1 = 2 *df*2 = 4,292	<0.001	0.108
	*SD*	15.13	16.21	12.65				
Gender	Male (*N*)	782	771	167				
	(%)	31.10%	58.10%	36.60%	266.27%	2	<0.001	
	Female (*N*)	1,735	557	289				
	(%)	68.90%	41.90%	63.40%				

The age and gender are comparable in the dataset from Germany and the Netherlands but the participants from China are significantly older, with a range of up to over 90 years, and have significantly more male participants. This may have to do with the fact that for both Germany and the Netherlands, but not in China, separate health services exist for patients older than 70 years.

The distributions of the PHQ-15 items within the three samples (Germany *N* = 2,517, China *N* = 1,329, Netherlands *N* = 456) are presented in Table 2.

**Table 2:** Descriptive statistics for the items of the PHQ15 for all three samples.

		**Germany (*N* = 2,517)**	**China (*N* = 1,329)**	**Netherlands (*N* = 456)**
**PHQ1** stomach pain	*Not bothered at all* *Bothered a little* *Bothered a lot*	*47.9 % 1,206* *25.9% 651* *26.2% 660*	*69.0% 917* *25.3% 336* *5.7% 76*	*39.1% 175* *42.6% 191* *18.3% 82*
**PHQ2** back pain	*Not bothered at all* *Bothered a little* *Bothered a lot*	*30.1% 757* *29.6% 746* *40.3% 1,014*	*55.2% 733* *36.7% 488* *8.1% 108*	*29.6% 135* *40.8% 186* *29.6% 135*
**PHQ3** pain in your arms, legs, or joint	*Not bothered at all* *Bothered a little* *Bothered a lot*	*32.2% 811* *28.3% 712* *39.5% 994*	*52.9% 703* *34.5% 458* *12.6% 168*	*26.5% 121* *36.8% 168* *36.6% 167*
**PHQ5** headaches	*Not bothered at all* *Bothered a little* *Bothered a lot*	*32.3% 814* *37.1% 935* *30.5% 768*	*65.4% 869* *30.1% 400* *4.5% 60*	*66.9% 305* *24.1% 110* *9.0% 41*
**PHQ6** chest pain	*Not bothered at all* *Bothered a little* *Bothered a lot*	*53.4% 1,344* *27.7% 698* *18.9% 650*	*64.0% 850* *30.8% 409* *5.3% 70*	*32.5% 148* *43.2% 197* *24.3% 111*
**PHQ7** dizziness	*Not bothered at all* *Bothered a little* *Bothered a lot*	*36.2% 912* *37.9% 955* *25.8% 650*	*57.0% 758* *38.3% 509* *4.7% 62*	*57.0% 260* *30.7% 140* *12.3% 56*
**PHQ8** fainting spells	*Not bothered at all* *Bothered a little* *Bothered a lot*	*84.3% 2,121* *10.2% 257* *5.5% 139*	*83.4% 1,109* *13.2% 175* *3.4% 45*	*41.0% 187* *40.4% 184* *18.6% 85*
**PHQ10** shortness of breath	*Not bothered at all* *Bothered a little* *Bothered a lot*	*46.0% 1,158* *31.9% 804* *22.1% 555*	*67.0% 891* *25.7% 342* *7.2% 96*	*50.4% 230* *36.2% 165* *13.4% 61*
**PHQ11** pain or problems during sexual intercourse	*Not bothered at all* *Bothered a little* *Bothered a lot*	*69.9% 1,751* *13.7% 345* *16.7% 421*	*82.7% 1,099* *13.8% 184* *3.5% 46*	*38.2% 174* *45.0% 205* *16.9% 77*
**PHQ12** constipation, loose bowels, or diarrhea	*Not bothered at all* *Bothered a little* *Bothered a lot*	*41.0% 1,033* *31.1% 783* *27.9% 701*	*50.6% 672* *39.3% 522* *10.2% 135*	*37.7% 172* *43.4% 198* *18.9% 86*
**PHQ13** nausea, gas, or indigestion	*Not bothered at all* *Bothered a little* *Bothered a lot*	*34.1% 858* *32.7% 822* *33.3% 837*	*51.5% 685* *39.3% 522* *9.2% 122*	*20.8% 95* *59.2% 270* *20.0% 91*
**PHQ14** feeling tired or having low energy	*Not bothered at all* *Bothered a little* *Bothered a lot*	*8.6% 216* *28.6% 720* *62.8% 1,581*	*36.0% 479* *50.8% 675* *13.2% 175*	*22.4% 102* *41.7% 190* *36.0% 164*
**PHQ15** trouble sleeping	*Not bothered at all* *Bothered a little* *Bothered a lot*	*12.5% 314* *25.9% 651* *61.7% 1,552*	*43.5% 578* *40.8% 542* *15.7% 209*	*7.0% 19* *18.1% 49* *74.8% 202*

*Only three categories of possible answers exist within the PHQ15*.

Some items (e.g., PHQ8 [fainting spells] and PHQ11 [pain or problems during sexual intercourse]) have a bottom or floor effects so a metric scale cannot be assumed.

### CFA models for the three samples taken together

First, the bifactor model from Witthöft et al. (22) consisting of a general symptom distress factor and four orthogonal symptom-specific factors was estimated within a multigroup design. Our Model showed an acceptable but not excellent model fit in the three samples taken together. The Chi-Square-Test of the model fit was 1850.013 (*df* = 214, *p* < 0.0001) with a Chi-Square contribution from the German data of 1081.462, China about 531.294 and the Netherlands 237.257. The RMSEA was 0.073 (90%C.I. = 0.070, 0.076). With a CFI of 0.928 a TLI of 0.921, the first model only has an acceptable fit but not a good model fit.

Hence, a second model was explored assuming that the factors might not be completely independent, allowing for correlations between the four symptom-specific factors to be unequal to zero, in which the four symptom-specific factors would not be orthogonal anymore. This second model was explored over the three groups taken together again.

The Chi-Square-Test of the model fit was 641.186 (*df* = 46, *p* < 0.0001). The RMSEA was about 0.055 (90%C.I. = 0.051, 0.059). With a CFI of 0.980, a TLI of 0.965, this model had a better fit but the MPlus results showed that the latent variable covariance matrix was not positive definite for the Chinese sample, not for the whole model. Hence, a satisfactory fit for the three samples taken together could not be established.

### CFA model for the separate datasets

We split the dataset into the three datasets or Germany, China, and the Netherlands, and searched some specific model per country and special sample within these countries.

#### Germany

Starting with the German dataset, we tried a model comparable to Witthöft et al. (22) but allowing for correlations between the four symptom-specific factors. The Chi-Square-Test of the fit for this final German model was 265.638 (*df* = 46, *p* < 0.0001). The RMSEA was about 0.044 (90%C.I. = 0.039, 0.049). With a CFI of 0.983, a TLI of 0.971. The model therefore fits in the German dataset. The only difference between Witthöft et al. (22) are the low but essential correlations between the factors. Without these correlations, the model would not fit. The results of this model are shown in Tables 2a,b. Almost all correlations between the four factors were significant. Two factors (Pain and Cardio) have a correlation of about −0.546; the others have a lower absolute value. The model is shown in Figure 1.

**Table 2a:** Fitting solution in the German sample.

	**General Factor (S.E.)**	**Factor PAIN (S.E.)**	**Factor GASTRO (S.E.)**	**Factor CARDIO (S.E.)**	**Factor FATIGUE (S.E.)**
PHQ1	0.466 (0.023)		0.415 (0.026)		
PHQ2	0.573 (0.025)	0.596 (0.045)			
PHQ3	0.509 (0.026)	0.626 (0.046)			
PHQ5	0.579 (0.020)		−0.058 (0.029)		
PHQ6	0.623 (0.019)	−0.015 (0.033)			
PHQ7	0.675 (0.023)			0.381 (0.045)	
PHQ8	0.577 (0.034)			0.514 (0.056)	
PHQ10	0.592 (0.020)			0.003 (0.038)	
PHQ11	0.377 (0.028)			−0.227 (0.041)	
PHQ12	0.465 (0.026)		0.667 (0.026)		
PHQ13	0.553 (0.025)		0.729 (0.028)		
PHQ14	0.592 (0.023)				0.479 (0.056)
PHQ15	0.507 (0.026)				0.743 (0.081)

**Table 2b:** Correlations between the factors within the German sample.

	**Factor GASTRO**	**Factor CARDIO**	**Factor FATIGUE**
Factor PAIN	−0.081 (0.052) *p* = 0.118	−0.546 (0.080) *p* < 0.001	0.065 (0.052) *p* = 0.209
Factor GASTRO		−0.247 (0.066) *p* < 0.001	0.171 (0.046) *p* < 0.001
Factor CARDIO			−0.305 (0.081) *p* < 0.001

#### The netherlands

For the dataset from the Netherlands, explorative testing and using the modification indices showed that only a little change for item 6 (chest pain) had to be done (see Tables 3a,b). In the original model, this item belongs to pain-related symptoms. For our model, this item fits better to the cardiopulmonary-related symptoms factor. The Chi-Square-Test of the fit for the final Dutch model was 53.624 (*df* = 46, *p* = 0.2051). The RMSEA was about 0.019 (90%C.I. = 0.000, 0.038; probability of RMSEA ≤ 0.05 is = 0.999). With a CFI of 0.998, a TLI of 0.997. The model is shown in Figure 2.

**Table 3a:** Fitting solution in the sample from the Netherlands.

	**General Factor (S.E.)**	**Factor PAIN (S.E.)**	**Factor GASTRO (S.E.)**	**Factor CARDIO (S.E.)**	**Factor FATIGUE (S.E.)**
PHQ1	0.449 (0.097)		0.704 (0.073)		
PHQ2	0.568 (0.082)	0.750 (0.142)			
PHQ3	0.684 (0.054)	0.370 (0.139)			
PHQ5	0.499 (0.055)		0.208 (0.097)		
PHQ6	0.810 (0.045)			0.158 (0.157)	
PHQ7	0.612 (0.096)			0.450 (0.138)	
PHQ8	0.665 (0.062)			0.268 (0.135)	
PHQ10	0.610 (0.093)			0.429 (0.140)	
PHQ11	0.339 (0.144)				
PHQ12	0.397 (0.084)		0.567 (0.071)		
PHQ13	0.390 (0.089)		0.630 (0.066)		
PHQ14	0.756 (0.053)				0.285 (0.153)
PHQ15	0.480 (0.079)				0.441 (0.142)

**Table 3b:** Correlations between the factors within the sample from the Netherlands.

	**Factor GASTRO**	**FCARDIO**	**FFATIGUE**
FPAIN	0.400 (0.172) *p* = 0.020	0.405 (0.206) *p* = 0.049	0.433 (0.244) *p* = 0.076
FGASTRO		0.519 (0.138) *p* < 0.001	0.444 (0.230) *p* = 0.054
FCARDIO			0.509 (0.292) *p* = 0.082

#### China

The structure from Witthöft et al. (22) could not be replicated in the dataset from China. We split the cardiopulmonary-related symptoms factor into two factors. These factors were correlated but estimate different latent variables. In the model for this dataset the pain-related symptoms factor includes items PHQ2 (back pain), PHQ3 (pain arm legs), and PHQ6 (chest pain), and the gastrointestinal-related symptoms factor includes items PHQ1 (stomach pain), PHQ5 (headaches), PHQ12 (constipation), and PHQ13 (nausea, gas). The first cardiopulmonary-related symptoms factor only includes items PHQ7 (dizziness) and PHQ10 (short breath), while the second cardiopulmonary-related symptoms factor is measured by PHQ6 (chest pain), PHQ8 (fainting spells), and PHQ11 (pain sexual intercourse) (see Tables 4a,b). The fatigue-related symptoms factor includes items PHQ14 and PHQ15. The Chi-Square-Test of the fit for the final Chinese model was 187.779 (*df* = 41, *p* < 0.0001). The RMSEA was about 0.052 (90%C.I. = 0.045, 0.060; probability of RMSEA ≤ 0.05 is = 0.325). A CFI of 0.977, a TLI of 0.957, show a good fit of the model, as shown in Figure 3.

**Table 4a:** Fitting solution in the Chinese sample.

	**General Factor (S.E.)**	**Factor PAIN (S.E.)**	**Factor GASTRO (S.E.)**	**Factor CARDIO1 (S.E.)**	**Factor CARDIO2 (S.E.)**	**Factor FATIGUE (S.E.)**
PHQ1	0.587 (0.041)		0.389 (0.060)			
PHQ2	0.532 (0.036)	0.620 (0.065)				
PHQ3	0.470 (0.033)	0.315 (0.051)				
PHQ5	0.872 (0.046)		−0.168 (0.074)			
PHQ6	0.587 (0.034)	0.301 (0.050)			0.254 (0.051)	
PHQ7	0.708 (0.033)			0.112 (0.066)		
PHQ8	0.532 (0.042)				0.547 (0.067)	
PHQ10	0.415 (0.044)			0.623 (0.184)		
PHQ11	0.443 (0.044)				0.443 (0.066)	
PHQ12	0.419 (0.046)		0.509 (0.047)			
PHQ13	0.498 (0.052)		0.656 (0.049)			
PHQ14	0.491 (0.048)					0.730 (0.046)
PHQ15	0.473 (0.038)					0.471 (0.043

**Table 4b:** Correlations between the factors within the Chinese sample.

	**Factor GASTRO**	**Factor CARDIO1**	**Factor CARDIO2**	**Factor FATIGUE**
Factor PAIN	0.337 (0.089) *p* < 0.001	0.447 (0.165) *p* = 0.007	−0.017 (0.122) *p* = 0.892	0.473 (0.078) *p* < 0.001
Factor GASTRO		0.407 (0.149) *p* = 0.006	0.217 (0.095) *p* = 0.003	0.619 (0.059) *p* < 0.001
Factor CARDIO1			0.585 (0.194) *p* = 0.003	0.668 (0.209) *p* = 0.001
Factor CARDIO2				−0.045 (0.116) *p* = 0.697

A part of the results from the different models—the thresholds between the points on the scale of the PHQ-15 items—can be interpreted and compared between the different countries. The results in Table 5 reflect the descriptive in Table 1 where the participants from the different samples respond differently. This diversity leads to different thresholds in the three samples. They can be deduced from Table 1 and are shown in the Table below. For example, in the PHQ-15 item 3 the thresholds for both European samples are nearly the same while the thresholds for the sample from China are substantially higher. However, for all used PHQ-15 items there are thresholds, which show that all three points of the used 3-point scale are useful. This is shown in Table 5.

**Table 5:** Thresholds between the different steps on the 3-point scale of the PHQ-15.

	**Germany threshold (S.E.)**	**China threshold (S.E.)**	**Netherlands threshold (S.E.)**
PHQ1 1 to 2	−0.052 (0.025)	0.496 (0.036)	−0.278 (0.060)
PHQ1 2 to3	0.637 (0.027)	1.579 (0.056)	0.904 (0.069)
PHQ2 1 to 2	−0.522 (0.026)	0.130 (0.034)	−0.536 (0.062)
PHQ2 2 to3	0.246 (0.025)	1.397 (0.050)	0.536 (0.062)
PHQ3 1 to 2	−0.462 (0.026)	0.073 (0.034)	−0.627 (0.069)
PHQ3 2 to3	0.267 (0.025)	1.144 (0.044)	0.342 (0.060)
PHQ5 1 to 2	−0.485 (0.026)	0.396 (0.035)	0.437 (0.061)
PHQ5 2 to3	0.510 (0.026)	1.694 (0.060)	1.341 (0.083)
PHQ6 1 to 2	0.085 (0.025)	0.357 (0.035)	−0.455 (0.061)
PHQ6 2 to3	0.883 (0.029)	1.619 (0.057)	0.695 (0.064)
PHQ7 1 to 2	−0.352 (0.026)	0.177 (0.035)	0.177 (0.059)
PHQ7 2 to3	0.649 (0.027)	1.678 (0.059)	1.161 (0.076)
PHQ8 1 to 2	1.005 (0.030)	0.972 (0.041)	−0.227 (0.059)
PHQ8 2 to3	1.596 (0.041)	1.827 (0.066)	0.891 (0.068)
PHQ10 1 to 2	−0.100 (0.025)	0.441 (0.036)	0.011 (0.059)
PHQ10 2 to3	0.771 (0.028)	1.459 (0.052)	1.109 (0.074)
PHQ11 1 to 2	0.512 (0.026)	0.942 (0.041)	−0.301 (0.060)
PHQ11 2 to3	0.965 (0.030)	1.817 (0.065)	0.959 (0.070)
PHQ12 1 to 2	−0.226 (0.025)	0.014 (0.034)	−0.313 (0.060)
PHQ12 2 to3	0.587 (0.027)	1.237 (0.047)	0.883 (0.068)
PHQ13 1 to 2	−0.410 (0.026)	0.039 (0.034)	−0.812 (0.066)
PHQ13 2 to3	0.433 (0.026)	1.330 (0.048)	0.843 (0.067)
PHQ14 1 to 2	−1.367 (0.036)	−0.357 (0.035)	−0.760 (0.065)
PHQ14 2 to3	−0.327 (0.025)	1.118 (0.043)	0.359 (0.060)
PHQ15 1 to 2	−1.152 (0.032)	−0.164 (0.035)	−1.473 (0.115)
PHQ15 2 to3	−0.297 (0.025)	1.006 (0.042)	−0.669 (0.083)

#### PHQ-9 and GAD-7

We then tested the predictive value of the PHQ-9 and the GAD-7 questionnaires within these models. Within the German and the Chinese sample the PHQ-9 had good prediction values for most PHQ-15 factors while the GAD-7 predicted the general factor and the fatigue factor well. In the sample from the Netherlands, only the general factor is well-predicted by the PHQ-9 and the GAD-7. This is shown in Table 6.

**Table 6:** Different factors predicted by PHQ-9 and GAD-7.

**Factor**	**Germany**				**China**				**Netherlands**			
	**PHQ-9**		**GAD**		**PHQ-9**		**GAD-7**		**PHQ-9**		**GAD-7**	
	**Est. (SE)**	***p***	**Est. (SE)**	***p***	**Est. (SE)**	***p***	**Est. (SE)**	***p***	**Est. (SE)**	***p***	**Est. (SE)**	***P***
General	**0.348 (0.027)**	**<0.001**	**0.433 (0.029)**	**<0.001**	**0.168 (0.043)**	**<0.001**	**0.211 (0.038)**	**<0.001**	**0.178 (0.059)**	**0.002**	**0.167 (0.061)**	**0.006**
Pain	0.027 (0.020)	0.171	0.019 (0.020)	0.331	**0.160 (0.068)**	**0.019**	0.108 (0.061)	0.080	0.000 (0.002)	0.980	0.035 (0.065)	0.595
Gastro	**0.057 (0.020)**	**0.004**	0.017 (0.015)	0.254	**0.206 (0.046)**	**<0.001**	**0.093 (0.035)**	**0.009**	0.036 (0.039)	0.357	0.010 (0.023)	0.648
Cardio	**0.085 (0.043)**	**0.049**	0.087 (0.057)	0.123	0.076 (0.047)	0.107	0.027 (0.027)	0.315	0.068 (0.056)	0.224	0.055 (0.058)	0.337
Fatigue	**0.763 (0.030)**	**<0.001**	**0.886 (0.035)**	**<0.001**	**0.402 (0.046)**	**<0.001**	**0.384 (0.047)**	**<0.001**	0.036 (0.074)	0.623	0.001 (0.004)	0.988

*Annotation: For this comparison we use only the second cardio factor within the sample from China. Significant regression weights are printed in bold*.

## Discussion

This study examines the factor structure of the PHQ-15 in a cross country and cross cultural setting involving China, with a total of 4302 participants. It shows that the factor structure found by Witthöft et al. (22) consisting of a general symptom distress factor and four orthogonal symptom-specific factors (pain-, gastrointestinal-, cardiopulmonary-, and fatigue-related symptoms) does not fit the full sample consisting of all three study populations.

An analysis per sample showed similarities with the Witthöft model in the German and the Dutch population. In the German sample the model of Witthöft could be replicated if low but essential correlations between the factors were allowed. We find a comparable but different model for the dataset from the Netherlands. The Dutch sample showed an excellent fit with the original model if item 6 was adapted belonging to pain-related symptoms toward fitting this item to the cardiopulmonary-related symptoms factor.

However, the Witthöft model could not be replicated in the Chinese sample. There were differences in pain, in the sense that the cardiovascular item chest pain was mostly reported in the context of general pain symptoms. Also, items like fainting, chest pain and problems with sexual intercourse were related, contrary to the European samples. Hence the Chinese sample seems to have different properties compared to the European samples. This may be due to epidemiological differences, such as that cardiovascular symptoms were reported and experienced in the context of sexual symptoms. But it might also be associated with cultural differences in body and illness perception, or in scripting, as described by Kleinman (64); a script concerning a specific clustering of symptoms may be shared by patients and Chinese mental health professionals and this might be one of such scripts. Ryder and Chentsova-Dutton (65) describe so-called “scripts”as … “organized units of culturally salient knowledge, such as knowledge about the ways in which one communicates distress… based on observation as well as formal learning. Scripts serve as mechanisms for rapid, automatic retrieval of information and recognition of patterns. …Once scripts are enacted they are observable by others as behavior and become elements of the larger cultural context” (66). In their elaboration, they refer to Kleinman and Kleinman who explain the meaning of scripts as potentially influenced by political events such as the Cultural Revolution. In this way, symptoms of distress i.e., depression could have dangerous connotations if expressed as such (67, 68) and thus lead to different scripts for distress (68).

Thus, cultural scripts (66) shape the ways in which people attend and react to particular experiences marked as important in some way. In some cases, pathological loops can form, where attention to a particular symptom can accentuate its severity and give rise to related symptoms' (65).

Another explanation may be fear of stigma; if sexual intercourse would be experienced as problematic, it might be reported in the context of symptoms suggesting cardiovascular disease, and not as standalone. A connection between shame and stigma has been reported for schizophrenia (69). The role of stigma in recognition of mental disorders in China has been explored (70). The literature indicates that higher rates of shyness and other normal interpersonal concerns in Chinese cultural contexts exist (71). Such shame may also play a role in sexual problems if it occurs in the context of unrecognized mental disorder, and thus in the reporting of physical symptoms in SSD.

Regarding a possible association between factor structure and health care settings, this was considered as a possibility because somatic symptoms may be perceived as providing more effective access to health care resources (38), however, this does not seem to be corroborated by the results. The German and Dutch samples seem to be congruent although from different health care settings, namely a general hospital setting and a specialized mental health care setting for SSD. The Chinese sample also concerns a general hospital setting, but the Chinese sample shows a different factorial structure from the European samples.

This different factorial structure in the Chinese sample does not seem to be explained by different associations with depression and anxiety either, as both the German and the Chinese sample showed that the PHQ-9 had good predictive values for most PHQ-15 factors while the GAD-7 well-predicted the general factor and the fatigue factor. In contrast, the sample from the Netherlands found the PHQ-9 and the GAD-7 to be only predictive for the PHQ-15 general factor.

The findings of this study suggest that the different factorial structure of the PHQ-15 in China is not to be explained by differences in concomitant depression or anxiety. Cultural factors such as stigma, scripting, or different concepts of disease (36), may play a role in this. Further research is needed to explore this, and replication studies are needed regarding the factorial structure of the PHQ-15 in China. Nevertheless, with this knowledge the PHQ15 can be applied in China, taking this small difference in somatic symptom clusters for China into account. Future studies should also explore to what extend the different factorial structures might bias data analysis when the PHQ-15 is used as a unidimensional measure. Furthermore, they should investigate whether any confounding factors could be identified which drive the differences between cultures.

### Limitations of the study

It can be considered a limitation of the study that health care settings and culture differences overlap and hence their assessment cannot be separated sufficiently to enable firm conclusions in this matter.

## Conclusion

The PHQ-15 is a valid questionnaire that can discern somatization from anxiety and depression within different cultures like Europe or China. The questionnaire can be fitted to a bifactorial model, however, we can only recommend the use of the general factor. Application of the orthogonal subscales in non-European samples is not corroborated by the results. Further research is needed to explore explanations and to replicate the findings of this study.

## Author contributions

Acquisition of the study was by KF. CvdF-C, KF, RL, and LdV designed the study. CvdF-C, LdV, LZ, YL, ZD, RS, SN, FF provided the data. RL, FF, CvdF-C, LdV, and KF designed the analyses and RL performed them. CvdF-C, LdV, and RS contributed to the interpretation of the data. RL, LdV, and CvdF-C wrote the manuscript and LZ, YL, ZD, RS, SN, and FF contributed to the manuscript for the Chinese and German samples. All authors critically revised and approved the final version of the article.

### Conflict of interest statement

The authors declare that the research was conducted in the absence of any commercial or financial relationships that could be construed as a potential conflict of interest.
